# Metabolic Phenotype Characterization of *Botrytis cinerea*, the Causal Agent of Gray Mold

**DOI:** 10.3389/fmicb.2018.00470

**Published:** 2018-03-13

**Authors:** Han-Cheng Wang, Li-Cui Li, Bin Cai, Liu-Ti Cai, Xing-Jiang Chen, Zhi-He Yu, Chuan-Qing Zhang

**Affiliations:** ^1^Guizhou Academy of Tobacco Science, Guiyang, China; ^2^Upland Flue-Cured Tobacco Quality and Ecology Key Laboratory of China Tobacco, Guiyang, China; ^3^College of Life Science, Yangtze University, Jingzhou, China; ^4^College of Agriculture and Food Science, Zhejiang Agriculture & Forestry University, Lin’an, China

**Keywords:** gray mold, biolog phenotype microarray, *Botrytis cinerea*, phenomics, metabolization

## Abstract

*Botrytis cinerea*, which causes gray mold, is an important pathogen in four important economic crops, tomato, tobacco, cucumber and strawberry, in China and worldwide. Metabolic phenomics data on *B. cinerea* isolates from these four crops were characterized and compared for 950 phenotypes with a BIOLOG Phenotype MicroArray (PM). The results showed that the metabolic fingerprints of the four *B. cinerea* isolates were similar to each other with minimal differences. *B. cinerea* isolates all metabolized more than 17% of the tested carbon sources, 63% of the amino acid nitrogen substrates, 80% of the peptide nitrogen substrates, 93% of the phosphorus substrates, and 97% of the sulfur substrates. Carbon substrates of organic acids and carbohydrates, and nitrogen substrates of amino acids and peptides were the significant utilization patterns for *B. cinerea*. Each *B. cinerea* isolate contained 94 biosynthetic pathways. These isolates showed a large range of adaptabilities and were still able to metabolize substrates in the presence of the osmolytes, including up to 6% potassium chloride, 10% sodium chloride, 5% sodium sulfate, 6% sodium formate, 20% ethylene glycol, and 3% urea. These isolates all showed active metabolism in environments with pH values from 3.5 to 8.5 and exhibited decarboxylase activities. These characterizations provide a theoretical basis for the study of *B. cinerea* in biochemistry and metabolic phenomics and provide valuable clues to finding potential new ways to manage gray mold.

## Introduction

Gray mold caused by *Botrytis* *cinerea* is a worldwide notorious disease affecting many important economic crops, such as tomato, cucumber, tobacco, strawberry, and lettuce (Coertze and Holz, 2002; Elmer and Michailides, 2007). More than 200 plant species are infected by this disease each year throughout the world (Williamson et al., 2007). Plant tissues of stems, leaves, flower petals, and berries could all be infected by *B. cinerea*. Losses frequently occur for both greenhouse-grown and field-grown crops (Pedras et al., 2011) and could exceed 40% if chemical control is not used (Villa-Rojas et al., 2012). Due to the broad economic impact of gray mold, *B.* *cinerea* is recognized as the second most important fungal pathogen (Dean et al., 2012). In China, four important crops (tomato, cucumber, strawberry, and tobacco) are grown throughout the country, and the losses caused by *B.* *cinerea* are enormous each year for these four crops (Wang et al., 2016a).

*Botrytis cinerea* is a necrotrophic pathogen and is more destructive on senescent and mature tissues (Williamson et al., 2007). It utilizes several mechanisms to kill host cells, including the stimulation of an oxidative burst, the secretion of cell wall-degrading enzymes, and the production of non-specific phytotoxic metabolites (botryoidal and botcinolides) (van Kan, 2006; Williamson et al., 2007; Amselem et al., 2011). These mechanisms might help this pathogen adapt itself to different tissues and conditions. Additionally, *B. cinerea* isolates differ in many biological characteristics, including their temperature sensitivity of mycelial growth and conidia death, their pH sensitivity of mycelial growth, their carbon and nitrogen sensitivity of both mycelial growth and conidia germination (Gao et al., 2009), and their pathogenicity (Kumari et al., 2014). In the last 20 years, *B. cinerea* has been studied in many crops during necrotrophic pathogenesis (van Kan, 2006; González-Fernández et al., 2014; Plesken et al., 2015). However, the metabolic basis for the no-host specificity of *B. cinerea* is poorly known, especially for these different isolates hosted from tomato, cucumber, strawberry and tobacco, which are four major economic crops in China and the world. Knowing the metabolic phenotype of *B. cinerea* will be valuable to understanding its biochemical properties and may also help to develop some potential measures to decrease the broad impact of gray mold on crops.

Traditionally, cellular metabolism pathways were characterized one at a time, which is time consuming and often qualitatively and vaguely defined. During the last 10 years, Biolog (Hayward, CA, United States) has developed a high-throughput phenotypic microarray (PM) system (OmniLog), which can characterize nearly 1000 metabolic phenotypes simultaneously (Bochner et al., 2001). This system provides an immediate sense of the phenotypic range of a microorganism and is much easier to operate (Bochner et al., 2001). In a simple run, PMs can characterize the use of carbon, nitrogen, sulfur, and phosphorus sources; the biosynthetic pathways; and the variations in osmotic pressure, ionic strength and pH. PMs have been utilized to analyze the phenotypes of many microorganisms, including *Bacillus subtilis* (Gusarov et al., 2009), *Escherichia coli* (Bochner et al., 2001), *Ralstonia solanacearum* (Chen et al., 2016), and *Alternaria alternata* (Wang et al., 2015b).

Therefore, the objective of this study was to (i) isolate and identify *B. cinerea* isolates from tomato, tobacco, cucumber, and strawberry and (ii) characterize the metabolic phenotype of *B. cinerea* hosted by these four different crops. The data provided by this study will be valuable to expanding the knowledge of the biochemistry and metabolic phenomics of *B. cinerea* isolates and will ideally assist in the development of more effective measures for gray mold management.

## Materials and Methods

### Origin and Collection of *B. cinerea* Isolates

In 2011 and 2014, during the disease epidemic season in Guizhou province of China, infected plant tissues with typical gray mold symptoms were sampled from tomato leaves, tobacco stems, cucumber flowers, and strawberry fruits. To isolate *B. cinerea* from these four hosts, infected tissues were cleaned with sterile water five times and then air-dried under laboratory conditions. Pieces of tissue (10 mm × 10 mm) were cut from the margins of the lesions, disinfected in a 0.05% NaClO (w/v) solution for 2 min, rinsed in sterile water three times, dried with sterile filter paper, and placed on potato dextrose agar (PDA; 200 g L^-1^ potato boiled for half an hour and strained, 20 g L^-1^ glucose, 16 g L^-1^ agar) medium (Sun et al., 2010). Plates were placed at 25°C in darkness for 4–5 days. Colonies with morphologies similar to that of *B. cinerea* were purified. Single-spore isolation was performed to obtain pure isolates from each crop. For conidia production, these isolates were incubated at 28°C for 7 days, and then plates were placed at 15°C for 48 h. Conidia were produced on the plates and collected with a sterile wet cotton swab. The conidia on the swab were washed with sterile water to obtain a final concentration of 1 × 10^5^ spores/ml for subsequent use.

### Morphological Identification of *B. cinerea*

Isolates of *B. cinerea* were grown on PDA plates, and conidia were produced as mentioned above. Fungal colonies, mycelia, conidia, and sclerotia of the pathogen were observed and verified with a microscope.

### Biolog Identification of *B. cinerea*

For the biological identification of suspect isolates, filamentous fungi inoculating fluid (containing 2.5 g L^-1^ Phytagel and 0.3 g L^-1^ Tween 40) (FF-IF, catalogue # 72106) and FF MicroPlate test panels (catalogue # 1006) were used (Wang et al., 2016b). All of these materials were all purchased from Biolog, Inc. (Hayward, CA, United States). Each isolate was identified according to the instructions of the Biolog system (Biolog, Inc., United States). The Biolog Microlog Fungal Identification System provides a phenotypic fingerprint of a microorganism that can be used to identify the organism at the species level.

### Molecular Identification of *B. cinerea*

Suspect isolates were retrieved on PDA plates at 25°C. After 4 days of incubation, fresh mycelia of each isolate were removed with a sterile needle and placed in a 2 ml Eppendorf tube. A TaKaRa MiniBEST Universal Genomic DNA Extraction Kit, Ver. 5.0 (Code No. 9765), was used to extract the DNA of each isolate according to the manufacturer’s instructions. For each suspect strain, polymerase chain reaction (PCR) using universal primers ITS-1 (5′-TCCGTAGGTGAACCTGCGG-3′) and ITS-4 (5′-TCCTCCGCTTATTGATATGC-3′) was performed to amplify the ITS1-5.8s-ITS2 region of rDNA, which yielded an approximately 500-bp product (Wang et al., 2011). The PCR conditions were as follows: 5 min at 95°C, 35 cycles of 60 s at 95 °C, 40 s at 55°C, and 90 s at 72°C, and a final extension at 72°C for 10 min. Products were verified on 1.0% agarose (Biowest^®^, Spain) gels at 254 nm (UV) and sequenced by Sangon Biotech (Shanghai) Co., Ltd.

### Phenotype Characterization

One isolate of *B. cinerea* from each crop was characterized to determine its phenotype by using the PM system (Biolog, Hayward, CA, United States) according to the published procedure (Bochner et al., 2001; von Eiff et al., 2006). All materials, media, and reagents for the PM system were purchased from Biolog. In total, 10 PM plates were utilized in this study. Plates 1–8 were used to assess the catabolic pathways of carbon (PM 1–2), nitrogen (PM 3, 6–8), phosphorus (PM 4), and sulfur (PM 4) and biosynthetic pathways (PM 5), and plates 9–10 were used for osmotic/ion (PM 9) and pH effects (PM 10). A conidia suspension of *B. cinerea* from each isolate was prepared as mentioned above and suspended in the appropriate medium containing sterile filamentous fungi inoculating fluid. To each well of the PM plates was added 100 μl of a cell suspension with 62% transmittance. FF-IF was utilized for PM plates 1 and 2. FF-IF plus 100 mmol/L D-glucose, 5 mmol/L potassium phosphate (pH 6.0), and 2 mmol/L sodium sulfate was used for plates 3, 5, 6, 7, and 8. FF-IF plus 100 mmol/L D-glucose was used for plate 4. FF-IF plus yeast nitrogen base and 100 mmol/L D-glucose was used for plates 9 and 10. Plates were placed in the OmniLog station at 28°C for 7 days. Phenotypic data were recorded every 15 min by capturing digital images of the microarrays and storing turbidity values. Data were analyzed using Kinetic and Parametric software (Biolog). Phenotypes were estimated according to the area of each well under the kinetic curve of dye formation. The experiment was repeated twice. Data from replicated experiments were combined for analysis, and the average phenotype characterization is presented. Heat maps of phenotype analysis was conducted with the software of HemI (Heatmap IIIlustrator, version 1.0) (Zhao et al., 2017).

## Results

### Isolation and Identification of *B. cinerea*

After incubation of the infected tissue pieces on PDA plates, a total of four single-spore isolates of *B. cinerea* were isolated, including one from tomato (V1), one from tobacco (T1), one from cucumber (C1), and one from strawberry (S1). All isolates exhibited the typical colony morphology on the test medium. Fungal colonies were initially colorless and turned gray to brown when the conidia were produced. Sclerotia produced in the culture were round or irregular. All four isolates were identified at the species level by the Biolog FF microplate. They were all species of *B. cinerea*. rDNA analysis of the ITS1-5.8s-ITS2 region revealed that all four isolates tested belonged to the same genus. Homology searches for fungal strains were conducted by BLAST analyses, which revealed that their sequences (GenBank Accession No. MG878387, MG878388, MG878389, and MG878390) presented 100% similarity with the sequences of *B. cinerea*.

Based on the morphology, Biolog identification analysis and sequences of the ITS1-5.8s-ITS2 region, all four isolates were confirmed to be *B. cinerea* Pers.:Fr.

### Phenotype Characterization

Using PM plates 1–10, the phenotypes of *B. cinerea* from the four hosts were characterized. In total, 950 different growth conditions were tested, including 190 carbon substrates, 95 nitrogen substrates, 285 nitrogen pathways, 59 phosphorus substrates, 35 sulfur substrates, 94 biosynthetic pathways, 96 osmotic and ionic conditions, and 96 pH environments. The metabolic fingerprints of *B. cinerea* hosted by tomato, tobacco, cucumber, and strawberry were very similar (**Supplementary Figure S1**). For carbon source metabolization by *B. cinerea*, the highest utilization ratio (25%) was found in isolate V1 from tomato, followed by isolate T1 from tobacco (24%), isolate C1 from cucumber (22%), and isolate S1 from strawberry (17%). For amino acid nitrogen substrate metabolization, the highest utilization ratio (80%) was found in isolate V1 from tomato, followed by isolate S1 from strawberry (75%), isolate C1 from cucumber (72%), and isolate T1 from tobacco (63%). For peptide nitrogen substrate metabolization, the highest utilization ratio (92%) was found in *B. cinerea* V1 from tomato, followed by S1 (89%), T1 (86%), and C1 (80%). For phosphorus substrate metabolization, the highest utilization ratio (100%) was found in T1, followed by C1 (98%), V1 (97%), and S1 (93%). For sulfur substrate metabolization, three isolates (T1, V1, and C1) of *B. cinerea* all exhibited 100% utilization, while isolate S1 exhibited 97% utilization. Meanwhile, for the biosynthetic pathways, all four tested isolates showed 100% utilization. For osmotic and ionic adaptability, *B. cinerea* could adapt to 91% of the tested conditions. In the pH environmental adaptability tests, the metabolization ratio for the four *B. cinerea* isolates ranged from 46 to 50%, and the highest ratio was found in T1 (50%), followed by V1 (49%), S1 (47%), and C1 (46%) (**Supplementary Table S1**).

**FIGURE 1:**
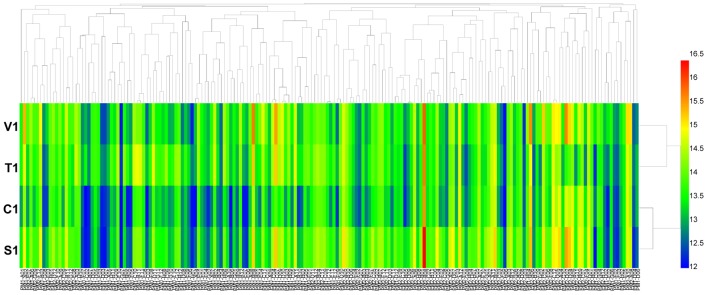
Overview of metabolic phenotypes of four isolates of *Botrytis cinerea* on 190 carbon (C) sources tested. V1, T1, C1, and S1 were the isolates of *B. cinerea* from tomato, tobacco, cucumber, and strawberry, respectively. Heatmap of maximum area values of 190 C sources expressed as maximum curve area monitored during 96 h of incubation. The legend of color code from blue to green, and red shades indicate low, moderate, and high utilization of C sources, respectively, assessed as arbitrary Omnilog values.

For carbon source (PM 1 and PM 2) utilization, *B. cinerea* was able to use more than 44 different carbon sources (**Figure 1**). Approximately 38 substrates were effectively metabolized by all four isolates of the pathogen, including L-arabinose, D-mannose, D-sorbitol, D-mannitol, glycerol, D-fructose, chondroitin sulfate C, and sucrose (**Supplementary Tables S2**, **S3**). In contrast, approximately 120 compounds could not be metabolized by the four tested isolates, including L-aspartic acid, D-saccharic acid, D-serine, succinic acid, and L-fucose (**Supplementary Tables S2**, **S3**). For these four isolates of *B. cinerea*, the carbon utilization level of several substrates was different, especially of L-proline, D-trehalose, dulcitol, D-ribose, adonitol, glycogen, laminarin, pectin, and D-melezitose (**Supplementary Tables S2**, **S3**). *B. cinerea* from tomato exhibited the highest carbon substrate utilization.

**FIGURE 2:**
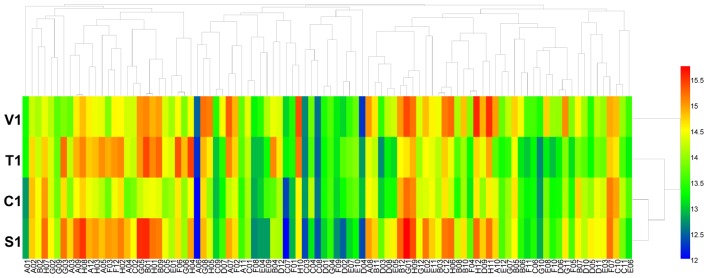
Overview of metabolic phenotypes of four isolates of *B. cinerea* on 95 nitrogen (N) sources tested. V1, T1, C1, and S1 were the isolates of *B. cinerea* from tomato, tobacco, cucumber, and strawberry, respectively. Heatmap of maximum area values of 95 N sources expressed as maximum curve area monitored during 96 h of incubation. The legend of color code from blue to green, and red shades indicate low, moderate, and high utilization of N sources, respectively, assessed as arbitrary Omnilog values.

Using the PM 3 plate, the four isolates of *B. cinerea* were characterized for their ability to metabolize 95 nitrogen substrates (amino acids). Approximately 55 substrates were effectively metabolized by the pathogen, including nitrite, ammonia, urea, nitrate, L-arginine, and L-alanine, while other substrates could not be metabolized or were poorly metabolized (**Figure 2** and **Supplementary Table S4**). For the four isolates of *B. cinerea*, nitrogen substrate utilization was different, especially of D-asparagine, L-aspartic acid, L-lysine, D-aspartic acid, methylamine, L-pyroglutamic acid, *N*-butylamine, *N*-amylamine, and formamide (**Supplementary Table S4**). The *B. cinerea* isolate from tomato exhibited the highest amino acid nitrogen substrate utilization.

**FIGURE 3:**
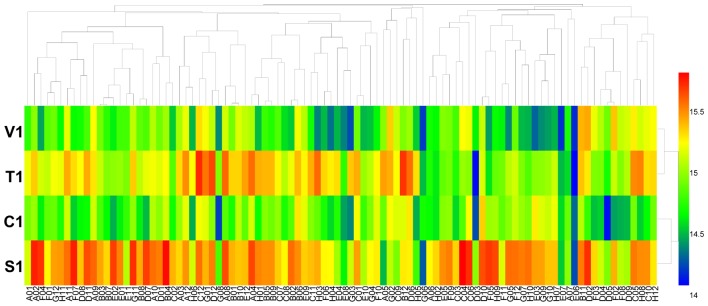
Overview of metabolic phenotypes of four isolates of *B. cinerea* on 59 phosphorous (P) sources and 35 sulfur (S) sources tested. V1, T1, C1, and S1 were the isolates of *B. cinerea* from tomato, tobacco, cucumber, and strawberry, respectively. Heatmap of maximum area values of 59 P and 35 S sources expressed as maximum curve area monitored during 96 h of incubation. The legend of color code from blue to green, and red shades indicate low, moderate, and high utilization of both P and S sources, respectively, assessed as arbitrary Omnilog values.

Using the PM 4 plate, the four isolates of *B. cinerea* were characterized for their ability to metabolize 59 phosphorus compounds (wells A2 to E12) and on 35 different sulfur substrates (wells F2 to H12). All four tested isolates of *B. cinerea* were very efficient in utilizing these two types of substrates (**Figure 3** and **Supplementary Table S5**). For phosphorus compounds, more than 55 compounds were effectively utilized. Isolate T1 from tobacco metabolized all tested phosphorus substrates; isolate V1 from tomato could utilize all tested phosphorus substrates except phosphono acetic acid (well E6) and 2-aminoethyl phosphonic acid (well E7); isolate C1 from cucumber could utilize all tested phosphorus substrates except dithiophosphate (well B2); and isolate S1 from strawberry could metabolize all tested phosphorus except tripoly-phosphate (well A5), phosphono acetic acid (well E6), 2-aminoethyl phosphonic acid (well E7), or methylene diphosphonic acid (well E8). For sulfur substrates, more than 34 were metabolized. *B. cinerea* isolates V1, T1, and C1 utilized all tested substrates, while isolate S1 could not utilize the substrate D,L-ethionine (well G6) (**Figure 3** and **Supplementary Table S5**).

Using the PM 5 plate, the 95 biosynthetic pathway test was carried out, and each *B. cinerea* isolate showed metabolization (**Supplementary Figure S1**). Using the PM 6, PM 7, and PM 8 plates (nitrogen pathways), *B. cinerea* metabolized more than 79.65% of the tested substrates (**Supplementary Figure S1**). The four isolates of *B. cinerea* showed similar metabolic fingerprints on plates PM 6, PM 7 and PM 8. Isolate C1 from cucumber could not metabolize Asp-Val (plate PM 6, well D3), Glu-Glu (plate PM 6, well D8), Gly-Gly (plate PM 6, well E5), or Gly-His (plate PM 6, well E6), while those compounds were utilized by the other three tested isolates of *B. cinerea*. Meanwhile, isolate C1 from cucumber could not utilize Gly-Ala (plate PM 6, well E2), Leu-Gly (plate PM 6, well H8), Gly-Gly-Leu (plate PM 8, well H5) or Gly-Phe-Phe (plate PM 8, well H8), while these substrates were metabolized by the other three isolates of *B. cinerea*.

**FIGURE 4:**
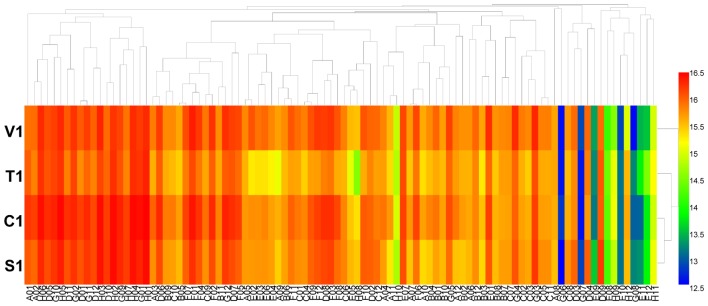
Overview of metabolic phenotypes of four isolates of *B. cinerea* at 96 osmotic and ionic (O) conditions tested. V1, T1, C1, and S1 were the isolates of *B. cinerea* from tomato, tobacco, cucumber, and strawberry, respectively. Heatmap of maximum area values of 96 O conditions expressed as maximum curve area monitored during 96 h of incubation. The legend of color code from blue to green, and red shades indicate low, moderate, and high metabolisation at O conditions, respectively, assessed as arbitrary Omnilog values.

**FIGURE 5:**
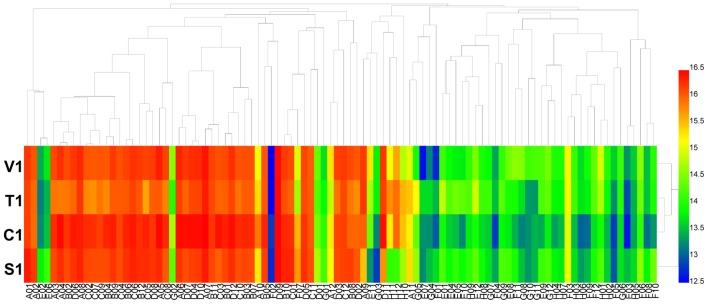
Overview of metabolic phenotypes of four isolates of *B. cinerea* at 96 pH environments tested. V1, T1, C1, and S1 were the isolates of *B. cinerea* from tomato, tobacco, cucumber, and strawberry, respectively. Heatmap of maximum area values of pH environments expressed as maximum curve area monitored during 96 h of incubation. The legend of color code from blue to green, and red shades indicate low, moderate, and high metabolisation at pH environments, respectively, assessed as arbitrary Omnilog values.

Using the PM 9 and PM 10 plates, the fungal growth under various stress conditions was tested. The results showed that the growth fingerprints of the four *B. cinerea* isolates under different stress conditions were similar. The isolates showed active metabolism, utilizing up to 6% potassium chloride, up to 10% sodium chloride, up to 5% sodium sulfate, up to 6% sodium formate, up to 20% ethylene glycol, up to 12% sodium lactate, up to 3% urea (except for isolate C1), up to 200 mM sodium phosphate (pH 7.0), up to 100 mM ammonium sulfate (pH 8.0), up to 20 mM sodium benzoate (pH 5.2) (except for the isolate C1), up to 100 mM sodium nitrite, and up to 100 mM sodium nitrate (**Figure 4** and **Supplementary Table S6**). When combined with different osmolytes under stress of 6% sodium chloride, *B. cinerea* grew effectively in all tests (plate PM 9, wells B1 to B12 and C1 to C12). The pH where most *B. cinerea* isolates grew effectively ranged from 3.5 to 8.5, with an optimal pH of approximately 7.0, while isolate S1 from strawberry continued to grow at pH 9. When combined with various amino acids under the stress of pH 4.5, *B. cinerea* grew effectively in most tests (plate PM 10, wells B1 to B12, C1 to C12, and D1 to D12), except when combined with the amino acid anthranilic acid (plate PM 10, well D1). In contrast, when combined with various amino acids under the stress of pH 9.5, the pathogen exhibited no growth (plate PM 10, wells E1 to E12, F1 to F12, and F1 to F12). When combined with different amino acids under the stress of pH 4.5 and pH 9.5, the decarboxylase and deaminase activities of the pathogen were tested on PM 10 in wells of B1-D12 and E1-G12, respectively. *B. cinerea* showed active decarboxylase activity but no deaminase activity (**Figure 5** and **Supplementary Table S7**). Moreover, the metabolic fingerprints of these four isolates of *B. cinerea* under stress from other compounds in the PM 10 plate (wells H1 to H12) were different. Isolates C1 and S1 could not grow under pressure from those stressors, while isolate V1 could grow when exposed to the following stressors: X-caprylate (plate PM 10, well H1), X-PO4 (plate PM 10, well H11) and X-SO4 (plate PM 10, well H12). Isolate T1 could grow when exposed to X-caprylate (plate PM 10, well H1), X-PO4 (plate PM 10, well H11), X-α-D-mannoside (plate PM 10, well H10), and X-SO4 (plate PM 10, well H12).

## Discussion

*Botrytis cinerea* is a destructive fungal pathogen distributed worldwide. Many molecular biological, genetic and genomic studies have been conducted on *B. cinerea* (Blanco et al., 2006; Kunz et al., 2006; Leroch et al., 2013; Kumari et al., 2014). Although this pathogen is commonly found in tomato, tobacco, strawberry, and cucumber hosts during the gray mold epidemic period (Coertze and Holz, 2002; Elmer and Michailides, 2007; Wang et al., 2011), the metabolic phenotypic diversity of this pathogen is still poorly understood. The PM system (Bochner et al., 2001) has received considerable attention in population studies of many microorganisms (Bochner, 2003; Viti et al., 2007). In this study, the metabolic ability of *B. cinerea* isolates obtained from tomato, tobacco, cucumber, and strawberry crops was systematically studied using PMs, and important metabolic diversity information was obtained.

*Botrytis cinerea* strains can infect more than 200 plant species worldwide (Williamson et al., 2007). Different crops normally have different nutrition substrates in their tissues, as well as different osmolytes and pH environments, which affect the survival and pathogenicity of pathogens (Fan et al., 2001; Lung et al., 2008). Regarding the crops used in our study, both tomato and tobacco belong to the family Solanaceae, cucumber belongs to Cucurbitaceae, and strawberry belongs to Rosaceae. Though the hosts of *B. cinerea* are different in their taxonomy, the metabolic phenotype characterization of *B. cinerea* isolates is quite similar, and only a small difference was found. Similar results from the biological characterization of the pathogen have also been found by *B. cinerea* isolates from tomato, pepper, strawberry, and grape have also been found (Gao et al., 2009). The number of carbon and nitrogen substrates utilized was highest for the *B. cinerea* isolate T1 from tomato. The reason for this difference is unclear and might be that tomato contains more carbon and nitrogen compounds than the other three crops. More work could be conducted to verify this hypothesis in the next study. Meanwhile, the numbers of osmolytes metabolized and pH conditions adapted to also different for these *B. cinerea*. This might be due to the differences in the osmolyte and pH conditions from which these *B. cinerea* isolates were sampled.

Our study showed that these four isolates of *B. cinerea* could metabolize a many of the carbon substrates and most of the nitrogen, sulfur, and phosphorus substrates. These data suggested that *B. cinerea* isolates from different crops might have some commonalities in their adaptability. The plates that were significantly metabolized were PM 1/PM 2, PM 3, PM 9, and PM 10. Similar findings have also been reported by other researchers studying other microorganisms (Friedl et al., 2008; Wang et al., 2015a). In our study, organic acids and carbohydrates for the carbon substrates and amino acids and peptides for the nitrogen substrates were significantly utilized by *B. cinerea*. These substrates are ubiquitously found in plant tissues and might support the survival of *B. cinerea* in different hosts and thus affect the pathogenicity of the pathogen. Additionally, the number of carbon substrates and nitrogen substrates utilized by *B. cinerea* was similar to that by the foliar fungal pathogen *Alternaria alternata* (Wang et al., 2015b) but much lower than that by the soil born pathogen *Phytophthora parasitica* (Wang et al., 2015c). This might be because the fact that nutrition substrates are abundant in the soil condition but relatively depleted in the upland tissues of plants. This hypothesis might be verified by future studies.

Additionally, although the osmolyte and pH conditions in tomato, tobacco, cucumber, and strawberry tissues are different, *B. cinerea* isolates from the four hosts presented similar metabolic fingerprints. The strong metabolic abilities of *B. cinerea* in various osmotic and pH conditions might help to support the great adaptability of the pathogen on different crops. At the lowest pH value of 3.5, all *B. cinerea* isolates exhibited effective growth, whereas no growth was observed at pH 9.5. This might be due to the pathogen preferring an acidic environment rather than an alkaline environment. Decarboxylases of the pathogen produce alkaline amines by metabolizing amino acids, which help to counteract an acidic pH. In contrast, a high pH can be counteracted by deaminases that produce acids (Durso et al., 2004; Maurer et al., 2005). In our study, *B. cinerea* isolates from different hosts all exhibited high decarboxylase activity but no deaminase activity. These results suggest that *B. cinerea* can adapt to the acidic tissue pH that occurs during the tissue development of a plant.

Numerous discoveries from analysis of the metabolic phenomics of microorganisms have been reported (Bochner et al., 2008; Gusarov et al., 2009), and their results have been widely utilized (Friedl et al., 2008). Some ideas related to novel approaches for gray mold control might be obtained from the phenomics of *B. cinerea* in this study. Enhancing the amount of carbon and nitrogen sources that cannot be used by *B. cinerea* or decreasing the amount of sources that can be utilized might affect the infection of this pathogen and subsequently depress the disease losses. In this study, *B. cinerea* could not grow with 4% urea, 50 mM sodium benzoate (pH 5.2) or pH >9.0. Thus, changing the pH or osmolyte content of host plants or the environment to make them unsuitable for *B. cinerea* may help to decrease its infection. However, the condition actually required to inhibit the growth of *B. cinerea* might be challenging to attain in a plant or the environment. More research could be performed to verify this hypothesis in the next study.

## Conclusion

Although the phenomics of *B. cinerea* from tomato, tobacco, cucumber, and strawberry hosts showed few differences, the results of this study still enriched our knowledge of the metabolic characterization of *B. cinerea*, particularly its ability to utilize nutritional substrates and its adaptability in different environments. This feature of the results has provided valuable clues to finding new methods of gray mold management.

## Author Contributions

Conceived and designed the experiments: H-CW and C-QZ. Performed the experiments: L-CL, BC, L-TC, and X-JC. Wrote and revised the paper: H-CW, Z-HY, and C-QZ. All authors approved the final version of the manuscript.

## Conflict of Interest Statement

The authors declare that the research was conducted in the absence of any commercial or financial relationships that could be construed as a potential conflict of interest.

## Supplementary Material

FIGURE S1 Data for the Biolog Phenotype MicroArray PM 1–10 plates for the four isolates of *Botrytis cinerea*. V1, T1, C1, and S1 were the isolates of *B. cinerea* from tomato, tobacco, cucumber, and strawberry, respectively. The PM 1 to PM 10 plates for each isolate are presented at the first row, first column; first row, second column; second row, first column; second row, second column; third row, first column; third row, second column; fourth row, first column; fourth row, second column; fifth row, first column; and fifth row, second column, respectively. Numbers 1–12 on the horizontal axis and letters A–H on the vertical axis denote the layout of the tested Biolog MicroPlate. Utilization by the isolates of *B. cinerea* from the four hosts is indicated by the green areas of the growth curve for each substrate.

TABLE S1 Metabolic ratio of four isolates of *Botrytis cinerea* on Biolog PM 1 to 10 MicroPlates.

TABLE S2 Metabolic profiling of *B. cinerea* hosted by tomato, tobacco, cucumber, and strawberry on the PM 1 MicroPlate.

TABLE S3 Metabolic profiling of *B. cinerea* hosted by tomato, tobacco, cucumber, and strawberry on the PM 2 MicroPlate.

TABLE S4 Metabolic profiling of *B. cinerea* hosted by tomato, tobacco, cucumber, and strawberry on the PM 3 MicroPlate.

TABLE S5 Metabolic profiling of *B. cinerea* hosted by tomato, tobacco, cucumber, and strawberry on the PM 4 MicroPlate.

TABLE S6 Metabolic profiling of *B. cinerea* hosted by tomato, tobacco, cucumber, and strawberry on the PM 9 MicroPlate.

TABLE S7 Metabolic profiling of *B. cinerea* hosted by tomato, tobacco, cucumber, and strawberry on the PM 10 MicroPlate.
