# Supplementary figures and images for: Metabolic Phenotype Characterization of Botrytis cinerea, the Causal Agent of Gray Mold
Source: Front Microbiol. 2018 Mar 13;9:470. doi: 10.3389/fmicb.2018.00470 (PMC5859374; doi:10.3389/fmicb.2018.00470)

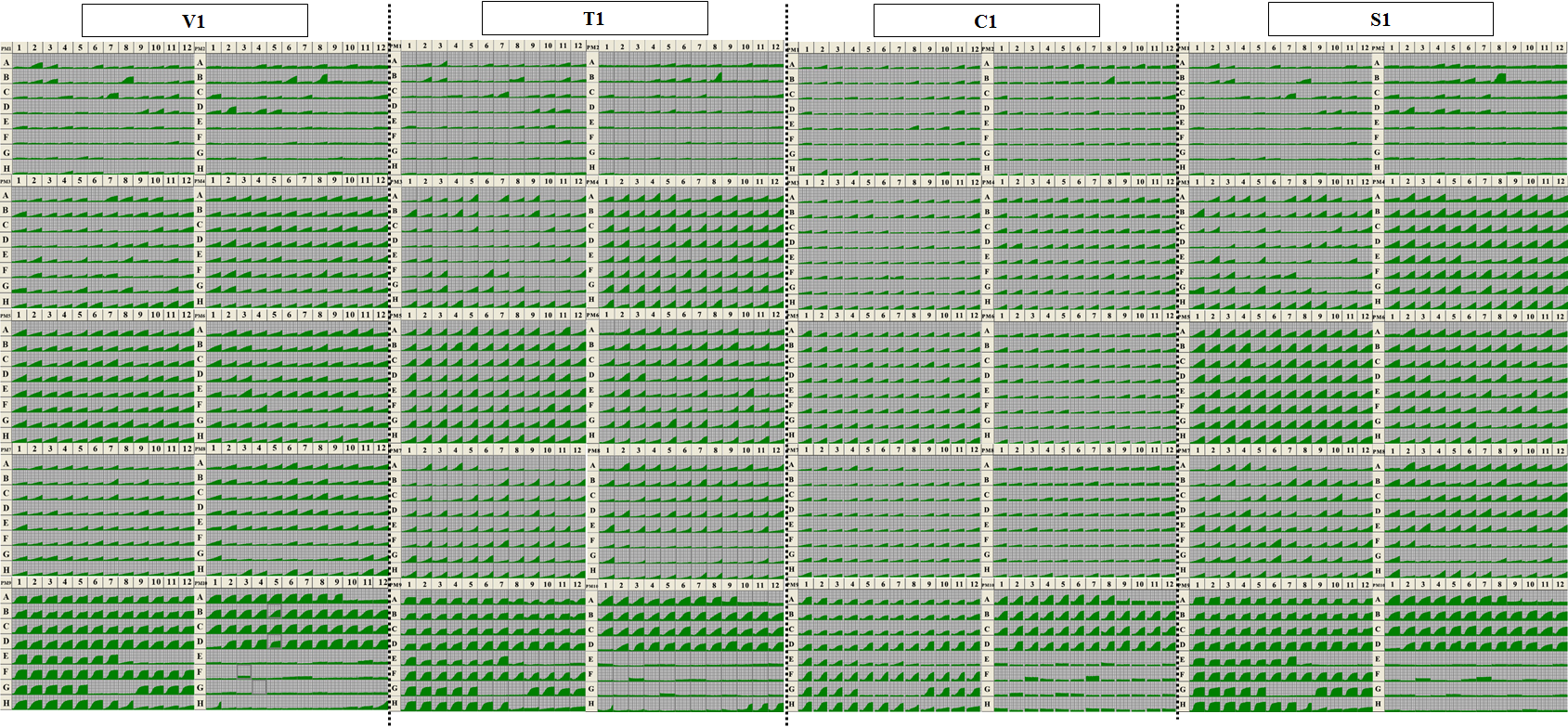

Supplement: FIGURE S1 — Data for the Biolog Phenotype MicroArray PM 1–10 plates for the four isolates of Botrytis cinerea. V1, T1, C1, and S1 were the isolates of B. cinerea from tomato, tobacco, cucumber, and strawberry, respectively. The PM 1 to PM 10 plates for each isolate are presented at the first row, first column; first row, second column; second row, first column; second row, second column; third row, first column; third row, second column; fourth row, first column; fourth row, second column; fifth row, first column; and fifth row, second column, respectively. Numbers 1–12 on the horizontal axis and letters A–H on the vertical axis denote the layout of the tested Biolog MicroPlate. Utilization by the isolates of B. cinerea from the four hosts is indicated by the green areas of the growth curve for each substrate. [file Image_1.TIF]
