# Supplementary material for: Metabolic Phenotype Characterization of Botrytis cinerea, the Causal Agent of Gray Mold
Source: Front Microbiol. 2018 Mar 13;9:470. doi: 10.3389/fmicb.2018.00470 (PMC5859374; doi:10.3389/fmicb.2018.00470)
Supplement: TABLE S1 — Metabolic ratio of four isolates of Botrytis cinerea on Biolog PM 1 to 10 MicroPlates. [file Table_1.DOC]

**Supplementary data**

**S1 Table**. Metabolic ratio of four isolates of *Botrytis cinerea* on Biolog PM 1 to 10 MicroPlates

| Isolates | | Host | Carbon substrate (%) ^x^ | Amino acid nitrogen substrates (%) | Peptide nitrogen substrates (%) | Phosphorus substrates (%) | Sulfur substrates (%) | Biosynthetic pathways (%) | Osmotic and ionic conditions (%) | pH condition (%) |
| --- | --- | --- | --- | --- | --- | --- | --- | --- | --- | --- |
| **V1** | tomato | | 26 | 80 | 92 | 97 | 100 | 100 | 93 | 49 |
| **T1** | tobacco | | 24 | 63 | 86 | 100 | 100 | 100 | 93 | 50 |
| **C1** | cucumber | | 221 | 72 | 80 | 98 | 100 | 100 | 91 | 46 |
| **S1** | strawberry | | 17 | 75 | 89 | 93 | 97 | 100 | 93 | 47 |

**^X^** Percentages listed in each column means that the number of substrates metabolized by *B. cinerea* in each kind of Biolog microplate divided by the total number of the substrates in each kind of Biolog microplate.

**S2 Table. Metabolic profiling of *Botrytis cinerea* hosted by tomato, tobacco, cucumber and strawberry on the PM 1 MicroPlate ***

| **#Cond.** | **Substrate** | **V1** | **T1** | **C1** | **S1** | **#Cond.** | **Substrate** | **V1** | **T1** | **C1** | **S1** | **#Cond.** | **Substrate** | **V1** | **T1** | **C1** | **S1** | **#Cond.** | **Substrate** | **V1** | **T1** | **C1** | **S1** |
| --- | --- | --- | --- | --- | --- | --- | --- | --- | --- | --- | --- | --- | --- | --- | --- | --- | --- | --- | --- | --- | --- | --- | --- |
| A1 | Negative Control | - | - | - | - | C1 | D-Glucose-6-Phosphate | - | - | - | - | E1 | L-Glutamine | + | + | + | + | G1 | Glycyl-L-Glutamic Acid | - | - | - | - |
| A2 | L-Arabinose | +++ | ++ | ++ | +++ | C2 | D-Galactonic Acid-γ-Lactone | - | - | - | - | E2 | M-Tartaric Acid | - | - | - | - | G2 | Tricarballylic Acid | - | - | - | - |
| A3 | N-Acetyl-D-Glucosamine | ++ | ++ | - | + | C3 | D,L-Malic Acid | - | - | - | - | E3 | D-Glucose-1-Phosphate | - | - | - | - | G3 | L-Serine | + | - | - | - |
| A4 | D-Saccharic Acid | - | - | - | - | C4 | D-Ribose | + | - | - | + | E4 | D-Fructose-6-Phosphate | + | - | - | - | G4 | L-Threonine | - | - | - | - |
| A5 | Succinic Acid | - | - | - | - | C5 | Tween20 | - | - | - | - | E5 | Tween80 | - | - | - | - | G5 | L-Alanine | + | + | - | + |
| A6 | D-Galactose | ++ | + | + | + | C6 | L-Rhamnose | + | + | - | + | E6 | α-Hydroxy Glutaric Acid-γ-Lactone | - | - | - | - | G6 | L-Alanyl-Glycine | - | - | - | - |
| A7 | L-Aspartic Acid | - | - | - | - | C7 | D-Fructose | +++ | +++ | + | +++ | E7 | α-Hydroxy Butyric Acid | - | - | - | - | G7 | Acetoacetic Acid | - | - | - | - |
| A8 | L-Proline | + | + | - | - | C8 | Acetic Acid | - | - | - | - | E8 | β-Methyl-DGlucoside | - | + | ++ | - | G8 | N-Acetyl-β-D-Mannosamine | - | - | - | - |
| A9 | D-Alanine | - | - | - | - | C9 | α-D-Glucose | + | + | + | + | E9 | Adonitol | - | + | - | - | G9 | MonoMethyl Succinate | - | - | - | - |
| A10 | D-Trehalose | + | + | - | - | C10 | Maltose | + | ++ | + | + | E10 | Maltotriose | + | + | + | + | G10 | Methyl Pyruvate | - | - | - | - |
| A11 | D-Mannose | + | ++ | ++ | + | C11 | D-Melibiose | ++ | ++ | + | + | E11 | 2-Deoxy-Adenosine | - | - | - | - | G11 | D-Malic Acid | - | - | - | - |
| A12 | Dulcitol | - | + | + | - | C12 | Thymidine | - | - | - | - | E12 | Adenosine | - | - | - | - | G12 | L-Malic Acid | - | - | - | - |
| B1 | D-Serine | - | - | - | - | D1 | L-Asparagine | - | - | + | - | F1 | Glycyl-L-Aspartic Acid | - | - | - | - | H1 | Glycyl-L-Proline | - | - | - | + |
| B2 | D-Sorbitol | ++ | ++ | + | ++ | D2 | D-Aspartic Acid | - | - | - | - | F2 | Citric Acid | - | - | - | - | H2 | p-Hydroxy PhenylAcetic Acid | - | - | ++ | - |
| B3 | Glycerol | ++ | ++ | + | ++ | D3 | D-Glucosaminic Acid | - | - | - | - | F3 | M-Inositol | - | - | - | - | H3 | m-Hydroxy PhenylAcetic Acid | - | - | - | - |
| B4 | L-Fucose | - | - | - | - | D4 | 1,2-Propanediol | - | - | - | - | F4 | D-Threonine | - | - | - | - | H4 | Tyramine | + | + | ++ | + |
| B5 | D-Glucuronic Acid | - | - | - | - | D5 | Tween40 | - | - | - | - | F5 | Fumaric Acid | - | - | - | - | H5 | D-Psicose | - | - | - | - |
| B6 | D-Gluconic Acid | - | - | - | - | D6 | α-Keto-Glutaric Acid | - | - | - | - | F6 | Bromo Succinic Acid | - | - | - | - | H6 | L-Lyxose | - | - | - | - |
| B7 | D,L-α-GlycerolPhosphate | - | - | - | - | D7 | α-Keto-Butyric Acid | - | - | - | - | F7 | Propionic Acid | - | - | - | - | H7 | Glucuronamide | - | - | - | - |
| B8 | D-Xylose | +++ | +++ | + | +++ | D8 | α-Methyl-D-Galactoside | - | - | - | - | F8 | Mucic Acid | - | - | - | - | H8 | Pyruvic Acid | - | - | - | - |
| B9 | L-Lactic Acid | - | - | - | - | D9 | α-D-Lactose | ++ | ++ | - | ++ | F9 | Glycolic Acid | - | - | - | - | H9 | L-Galactonic Acid-γ-Lactone | + | - | - | - |
| B10 | Formic Acid | - | - | - | - | D10 | Lactulose | ++ | ++ | - | ++ | F10 | Glyoxylic Acid | - | - | - | - | H10 | D-Galacturonic Acid | + | + | + | - |
| B11 | D-Mannitol | + | ++ | ++ | + | D11 | Sucrose | + | ++ | ++ | + | F11 | D-Cellobiose | + | ++ | + | + | H11 | Phenylethylamine | - | - | - | - |
| B12 | L-Glutamic Acid | + | + | + | - | D12 | Uridine | - | - | - | - | F12 | Inosine | - | - | - | - | H12 | 2-Aminoethanol | - | - | - | - |

*: #Cond. stands for the assay conducted on the Biolog PM 1 plate. “−”, “+”, “++” and “+++” indicate that *B. cinerea* could not utilize the tested substrate or utilized the substrate poorly, moderately or effectively, respectively, on the Biolog PM 1 MicroPlate after 7 days of incubation at 28 °C. “V1”, “T1”, “C1” and “S1” denote the isolates of *B. cinerea* hosted by tomato, tobacco, cucumber and strawberry, respectively.

**S3 Table. Metabolic profiling of *Botrytis cinerea* hosted by tomato, tobacco, cucumber and strawberry on the PM 2 MicroPlate ***

| **#Cond.** | **Substrate** | **V1** | **T1** | **C1** | **S1** | **#Cond.** | **Substrate** | **V1** | **T1** | **C1** | **S1** | **#Cond.** | **Substrate** | **V1** | **T1** | **C1** | **S1** | **#Cond.** | **Substrate** | **V1** | **T1** | **C1** | **S1** |
| --- | --- | --- | --- | --- | --- | --- | --- | --- | --- | --- | --- | --- | --- | --- | --- | --- | --- | --- | --- | --- | --- | --- | --- |
| A1 | Negative Control | - | - | - | - | C1 | Gentiobiose | - | - | - | - | E1 | Capric Acid | + | + | + | + | G1 | Acetamide | - | - | - | - |
| A2 | Chondroitin Sulfate C | +++ | ++ | ++ | +++ | C2 | L-Glucose | - | - | - | - | E2 | Caproic Acid | - | - | - | - | G2 | L-Alaninamide | - | - | - | - |
| A3 | α-Cyclodextrin | ++ | ++ | - | + | C3 | Lactitol | - | - | - | - | E3 | Citraconic Acid | - | - | - | - | G3 | N-Acetyl-LGlutamic Acid | + | - | - | - |
| A4 | β-Cyclodextrin | - | - | - | - | C4 | D-Melezitose | + | - | - | + | E4 | Citramalic Acid | + | - | - | - | G4 | L-Arginine | - | - | - | - |
| A5 | γ-Cyclodextrin | - | - | - | - | C5 | Maltitol | - | - | - | - | E5 | D-Glucosamine | - | - | - | - | G5 | Glycine | + | + | - | + |
| A6 | Dextrin | ++ | + | + | + | C6 | α-Methyl-D-Glucoside | + | + | - | + | E6 | 2-Hydroxy Benzoic Acid | - | - | - | - | G6 | L-Histidine | - | - | - | - |
| A7 | Gelatin | - | - | - | - | C7 | β-Methyl-D-Galactoside | +++ | +++ | + | +++ | E7 | 4-Hydroxy Benzoic Acid | - | - | - | - | G7 | L-Homoserine | - | - | - | - |
| A8 | Glycogen | + | + | - | - | C8 | 3-Methyl-Glucose | - | - | - | - | E8 | β-Hydroxy Butyric Acid | - | + | ++ | - | G8 | Hydroxy-L-Proline | - | - | - | - |
| A9 | Inulin | - | - | - | - | C9 | β-Methyl-D-Glucuronic Acid | + | + | + | + | E9 | γ-Hydroxy Butyric Acid | - | + | - | - | G9 | L-Isoleucine | - | - | - | - |
| A10 | Laminarin | + | + | - | - | C10 | α-Methyl-D-Mannoside | + | ++ | + | + | E10 | α-KetoValeric Acid | + | + | + | + | G10 | L-Leucine | - | - | - | - |
| A11 | Mannan | + | ++ | ++ | + | C11 | β-Methyl-D-Xyloside | ++ | ++ | + | + | E11 | Itaconic Acid | - | - | - | - | G11 | L-Lysine | - | - | - | - |
| A12 | Pectin | - | + | + | - | C12 | Palatinose | - | - | - | - | E12 | 5-Keto-D-Gluconic Acid | - | - | - | - | G12 | L-Methionine | - | - | - | - |
| B1 | N-Acetyl-D-Galactosamine | - | - | - | - | D1 | D-Raffinose | - | - | + | - | F1 | D-Lactic Acid MethylEster | - | - | - | - | H1 | L-Ornithine | - | - | - | + |
| B2 | N-Acetyl-Neuraminic Acid | ++ | ++ | + | ++ | D2 | Salicin | - | - | - | - | F2 | Malonic Acid | - | - | - | - | H2 | L-Phenylalanine | - | - | ++ | - |
| B3 | β-D-Allose | ++ | ++ | + | ++ | D3 | Sedoheptulosa | - | - | - | - | F3 | Melibionic Acid | - | - | - | - | H3 | L-Pyroglutamic Acid | - | - | - | - |
| B4 | Amygdalin | - | - | - | - | D4 | L-Sorbose | - | - | - | - | F4 | Oxalic Acid | - | - | - | - | H4 | L-Valine | + | + | ++ | + |
| B5 | D-Arabinose | - | - | - | - | D5 | Stachyose | - | - | - | - | F5 | Oxalomalic Acid | - | - | - | - | H5 | D,L-Carnitine | - | - | - | - |
| B6 | D-Arabitol | - | - | - | - | D6 | D-Tagatose | - | - | - | - | F6 | Quinic Acid | - | - | - | - | H6 | Sec-Butylamine | - | - | - | - |
| B7 | L-Arabitol | - | - | - | - | D7 | Turanose | - | - | - | - | F7 | D-Ribono-1,4-Lactone | - | - | - | - | H7 | D.L-Octopamine | - | - | - | - |
| B8 | Arbutin | +++ | +++ | + | +++ | D8 | Xylitol | - | - | - | - | F8 | Sebacic Acid | - | - | - | - | H8 | Putrescine | - | - | - | - |
| B9 | 2-Deoxy-DRibose | - | - | - | - | D9 | N-Acetyl-DGlucosaminitol | ++ | ++ | - | ++ | F9 | Sorbic Acid | - | - | - | - | H9 | Dihydroxy Acetone | + | - | - | - |
| B10 | I-Erythritol | - | - | - | - | D10 | γ-Amino Butyric Acid | ++ | ++ | - | ++ | F10 | Succinamic Acid | - | - | - | - | H10 | 2,3-Butanediol | + | + | + | - |
| B11 | D-Fucose | + | ++ | ++ | + | D11 | δ-AminoValeric Acid | + | ++ | ++ | + | F11 | D-Tartaric Acid | + | ++ | + | + | H11 | 2,3-Butanone | - | - | - | - |
| B12 | 3-0-β-D-Galacto-pyranosyl-D-Arabinose | + | + | + | - | D12 | Butyric Acid | - | - | - | - | F12 | L-Tartaric Acid | - | - | - | - | H12 | 3-Hydroxy2-Butanone | - | - | - | - |

*: #Cond. stands for the assay conducted on the Biolog PM 2 plate. “−”, “+”, “++” and “+++” indicate that *B. cinerea* could not utilize the tested substrate or utilized the substrate poorly, moderately or effectively, respectively, on the Biolog PM 2 MicroPlate after 7 days of incubation at 28 °C. “V1”, “T1”, “C1” and “S1” denote the isolates of *B. cinerea* hosted by tomato, tobacco, cucumber and strawberry, respectively.

**S4 Table. Metabolic profiling of *Botrytis cinerea* hosted by tomato, tobacco, cucumber and strawberry on the PM 3 MicroPlate ***

| **#Cond.** | **Substrate** | **V1** | **T1** | **C1** | **S1** | **#Cond.** | **Substrate** | **V1** |  | **T1** | **C1** | **S1** | **#Cond.** | **Substrate** | **V1** | **T1** | **C1** | **S1** | **#Cond.** | **Substrate** | **V1** | **T1** | **C1** | **S1** |
| --- | --- | --- | --- | --- | --- | --- | --- | --- | --- | --- | --- | --- | --- | --- | --- | --- | --- | --- | --- | --- | --- | --- | --- | --- |
| A1 | Negative Control | - | - | - | - | C1 | L-tyrosine | + |  | + | + | + | E1 | Histamine | + | ++ | + | + | G1 | Xanthine | + | ++ | +++ | +++ |
| A2 | Ammonia | + | ++ | ++ | +++ | C2 | L-valine | + |  | + | + | ++ | E2 | ß-phenylethyl-amine | + | ++ | + | +++ | G2 | Xanthosine | + | + | ++ | ++ |
| A3 | Nitrite | + | ++ | ++ | +++ | C3 | D-alanine | - |  | - | - | - | E3 | Tyramine | + | + | + | ++ | G3 | Uric acid | + | +++ | - | +++ |
| A4 | Nitrate | + | ++ | ++ | +++ | C4 | D-asparagine | + |  | - | + | - | E4 | Acetamide | + | - | - | - | G4 | Alloxan | + | + | + | - |
| A5 | Urea | + | +++ | ++ | +++ | C5 | D-aspartic acid | + |  | + | - | ++ | E5 | Formamide | + | - | - | +++ | G5 | Allantoin | + | ++ | ++ | +++ |
| A6 | Bluret | - | - | - | - | C6 | D-glutamic acid | + |  | - | + | + | E6 | Glucuronamide | - | - | + | - | G6 | Parabanic acid | + | ++ | ++ | ++ |
| A7 | L-alanine | +++ | +++ | ++ | +++ | C7 | D-lysine | - |  | - | - | - | E7 | D,L-lactamide | - | + | - | - | G7 | D,L-α-amino-N-butyric acid | + | + | ++ | + |
| A8 | L-arginine | ++ | ++ | ++ | +++ | C8 | D-serine | - |  | - | - | - | E8 | D-glucosamine | + | - | + | + | G8 | γ-amino-N-butyric acid | + | ++ | ++ | ++ |
| A9 | L-asparagine | ++ | +++ | ++ | +++ | C9 | D-valine | - |  | - | - | - | E9 | D-galactosamine | + | - | - | - | G9 | ε-amino-N-caproic acid | + | + | + | + |
| A10 | L-aspartic acid | ++ | - | ++ | +++ | C10 | L-citrulline | + |  | ++ | + | +++ | E10 | D-mannosamine | - | + | - | - | G10 | D,L-α-amino-caprylic acid | - | - | - | - |
| A11 | L-cysteine | - | - | - | - | C11 | L-homoserine | + |  | - | + | + | E11 | N-acetyl-D-glucosamine | + | ++ | + | +++ | G11 | δ-amino-N-valeric acid | + | + | ++ | ++ |
| A12 | L-glutamic acid | + | +++ | ++ | ++ | C12 | L-ornithine | + |  | ++ | ++ | +++ | E12 | N-acetyl-D-galactosamine | - | - | - | - | G12 | α-amino-N-valeric acid | + | - | ++ | + |
| B1 | L-glutamine | ++ | +++ | ++ | +++ | D1 | N-acetyl-D,L-glutamic acid | - |  | - | + | - | F1 | N-acetyl-D-mannosamine | - | - | + | - | H1 | Ala-asp | + | +++ | ++ | +++ |
| B2 | Glycine | + | ++ | + | ++ | D2 | N-phthaloyl-L-glutamic Acid | - |  | - | + | - | F2 | Adenine | + | ++ | + | ++ | H2 | Ala-gln | + | +++ | ++ | +++ |
| B3 | L-histidine | + | ++ | + | ++ | D3 | L-pyroglutamic acid | + |  | - | - | + | F3 | Adenosine | + | ++ | + | +++ | H3 | Ala-glu | + | +++ | ++ | +++ |
| B4 | L-isoleucine | + | +++ | ++ | ++ | D4 | Hydroxylamine | - |  | - | - | - | F4 | Cytidine | + | - | - | + | H4 | Ala-gly | + | +++ | ++ | +++ |
| B5 | L-leucine | + | ++ | ++ | ++ | D5 | Methylamine | + |  | - | - | ++ | F5 | Cytosine | + | - | - | + | H5 | Ala-his | + | ++ | ++ | ++ |
| B6 | L-lysine | + | - | - | - | D6 | N-amylamine | + |  | - | - | + | F6 | Guanine | + | +++ | ++ | ++ | H6 | Ala-leu | + | ++ | ++ | +++ |
| B7 | L-methionine | + | + | ++ | ++ | D7 | N-butylamine | + |  | - | + | + | F7 | Guanosine | + | ++ | + | ++ | H7 | Ala-thr | + | +++ | ++ | +++ |
| B8 | L-phenylalanine | + | + | ++ | ++ | D8 | Ethylamine | + |  | - | + | +++ | F8 | Thymine | - | - | - | - | H8 | Gly-asn | + | +++ | ++ | +++ |
| B9 | L-proline | + | +++ | ++ | +++ | D9 | Ethanolamine | + |  | ++ | + | +++ | F9 | Thymidine | - | - | - | - | H9 | Gly-gln | + | +++ | ++ | +++ |
| B10 | L-serine | + | + | + | +++ | D10 | Ethylenediamine | + |  | - | - | ++ | F10 | Uracil | - | - | - | - | H10 | Gly-glu | + | +++ | ++ | ++ |
| B11 | L-threonine | + | + | + | ++ | D11 | Putrescine | + |  | + | + | ++ | F11 | Uridine | - | - | - | - | H11 | Gly-met | + | +++ | ++ | +++ |
| B12 | L-tryptophan | + | + | +++ | +++ | D12 | Agmatine | + |  | ++ | + | + | F12 | Inosine | + | +++ | ++ | ++ | H12 | Met-ala | + | ++ | ++ | +++ |

*: #Cond. stands for the assay conducted on the Biolog PM 3 plate. “−”, “+”, “++” and “+++” indicate that *B. cinerea* could not utilize the tested substrate or utilized the substrate poorly, moderately or effectively, respectively, on the Biolog PM 3 MicroPlate after 7 days of incubation at 28 °C. “V1”, “T1”, “C1” and “S1” denote the isolates of *B. cinerea* hosted by tomato, tobacco, cucumber and strawberry, respectively.

**S5 Table. Metabolic profiling of *Botrytis cinerea* hosted by tomato, tobacco, cucumber and strawberry on the PM 4 MicroPlate ***

| **#Cond.** | **Substrate** | **V1** | **T1** | **C1** | **S1** | **#Cond.** | **Substrate** | **V1** |  | **T1** | **C1** | **S1** | **#Cond.** | **Substrate** | **V1** | **T1** | **C1** | **S1** | **#Cond.** | **Substrate** | **V1** | **T1** | **C1** | **S1** |
| --- | --- | --- | --- | --- | --- | --- | --- | --- | --- | --- | --- | --- | --- | --- | --- | --- | --- | --- | --- | --- | --- | --- | --- | --- |
| A1 | Negative Control | - | - | - | - | C1 | Phosphoenol pyruvate | ++ | +++ | ++ | +++ | ++ | E1 | O-phospho-D-tyrosine | ++ | +++ | ++ | +++ | G1 | N-acetyl-L-cysteine | ++ | +++ | ++ | +++ |
| A2 | Phosphate | ++ | +++ | ++ | +++ | C2 | Phospho-glycolic acid | ++ | +++ | ++ | +++ | ++ | E2 | O-phospho-L-tyrosine | ++ | +++ | ++ | +++ | G2 | S-methyl-L-cysteine | ++ | +++ | ++ | +++ |
| A3 | Pyrophosphate | ++ | +++ | ++ | +++ | C3 | D-glucose-1-phosphate | ++ | +++ | ++ | +++ | ++ | E3 | Phosphocreatine | ++ | +++ | ++ | +++ | G3 | Cystathionine | ++ | +++ | ++ | +++ |
| A4 | Trimeta-phosphate | ++ | +++ | ++ | +++ | C4 | D-glucose-6-phosphate | ++ | +++ | ++ | +++ | ++ | E4 | Phosphoryl choline | ++ | +++ | ++ | +++ | G4 | Lanthionine | ++ | +++ | ++ | +++ |
| A5 | Tripoly-phosphate | ++ | +++ | ++ | - | C5 | 2-deoxy-D-glucose 6-phosphate | ++ | +++ | ++ | +++ | ++ | E5 | O-phosphoryl-ethanolamine | ++ | +++ | ++ | +++ | G5 | Glutathione | ++ | +++ | ++ | +++ |
| A6 | Triethyl phosphate | ++ | + | ++ | + | C6 | D-glucosamine-6-phosphate | ++ | +++ | ++ | +++ | ++ | E6 | Phosphono acetic acid | - | + | ++ | - | G6 | D,L-ethionine | ++ | ++ | + | - |
| A7 | Hypophosphite | ++ | + | ++ | + | C7 | 6-phospho-gluconic acid | ++ | +++ | ++ | +++ | ++ | E7 | 2-aminoethyl phosphonic acid | - | +++ | ++ | - | G7 | L-methionine | ++ | +++ | ++ | +++ |
| A8 | Adenosine-2’-monophosphate | ++ | +++ | ++ | +++ | C8 | Cytidine-2’-monophosphate | ++ | +++ | ++ | +++ | ++ | E8 | Methylene diphosphonic acid | + | +++ | ++ | - | G8 | D-Methionine | ++ | +++ | ++ | +++ |
| A9 | Adenosine-3’-monophosphate | ++ | +++ | ++ | +++ | C9 | Cytidine-3’-monophosphate | ++ | +++ | ++ | +++ | ++ | E9 | Thymidine-3’-monophosphate | ++ | +++ | ++ | +++ | G9 | Glycyl-L-methionine | ++ | +++ | ++ | +++ |
| A10 | Adenosine-5’-monophosphate | ++ | +++ | ++ | +++ | C10 | Cytidine-5’-monophosphate | ++ | +++ | ++ | +++ | ++ | E10 | Thymidine-5’-monophosphate | ++ | +++ | ++ | +++ | G10 | N-acetyl-D,L-methionine | ++ | +++ | ++ | +++ |
| A11 | Adenosine-2’,3’-cyclic monophosphate | ++ | +++ | ++ | +++ | C11 | Cytidine-2’,3’-cyclic monophosphate | ++ | +++ | ++ | +++ | ++ | E11 | Inositol hexaphosphate | ++ | +++ | ++ | +++ | G11 | L-Methionine sulfoxide | ++ | +++ | ++ | +++ |
| A12 | Adenosine-3’,5’-cyclic monophosphate | ++ | +++ | ++ | +++ | C12 | Cytidine-3’,5’-cyclic monophosphate | ++ | +++ | ++ | +++ | ++ | E12 | Thymidine 3’,5’-cyclic monophosphate | ++ | +++ | ++ | +++ | G12 | L-methionine sulfone | ++ | +++ | ++ | +++ |
| B1 | Thiophosphate | ++ | +++ | ++ | +++ | D1 | D-mannose-1-phosphate | ++ | +++ | ++ | +++ | ++ | F1 | Negative control | ++ | +++ | ++ | +++ | H1 | L-djenkolic acid | ++ | +++ | ++ | +++ |
| B2 | Dithiophosphate | ++ | +++ | - | +++ | D2 | D-mannose-6-phosphate | ++ | +++ | ++ | +++ | ++ | F2 | Sulfate | ++ | +++ | ++ | +++ | H2 | Thiourea | ++ | ++ | ++ | +++ |
| B3 | D,L-α-Glycerol phosphate | ++ | +++ | ++ | +++ | D3 | Cysteamine-S-phosphate | ++ | + | + | +++ | ++ | F3 | Thiosulfate | ++ | +++ | ++ | +++ | H3 | 1-thio-β-D-glucose | ++ | +++ | ++ | +++ |
| B4 | β-Glycerol phosphate | ++ | +++ | ++ | +++ | D4 | Phospho-L-arginine | ++ | +++ | ++ | +++ | ++ | F4 | Tetrathionate | ++ | +++ | ++ | +++ | H4 | D,L-lipoamide | ++ | +++ | ++ | +++ |
| B5 | Carbamyl phosphate | ++ | +++ | ++ | +++ | D5 | O-phospho-D-serine | ++ | +++ | ++ | +++ | ++ | F5 | Thiophosphate | ++ | +++ | ++ | +++ | H5 | Taurocholic acid | ++ | +++ | ++ | +++ |
| B6 | D-2-phospho-glyceric acid | ++ | +++ | ++ | +++ | D6 | O-phospho-L-serine | ++ | +++ | ++ | +++ | ++ | F6 | Dithiophosphate | ++ | +++ | ++ | +++ | H6 | Taurine | ++ | +++ | ++ | +++ |
| B7 | D-3-phospho-glyceric acid | ++ | +++ | ++ | +++ | D7 | O-phospho-L-threonine | ++ | +++ | ++ | +++ | ++ | F7 | L-cysteine | ++ | +++ | ++ | +++ | H7 | Hypotaurine | ++ | +++ | ++ | +++ |
| B8 | Guanosine-2’-monophosphate | ++ | +++ | ++ | +++ | D8 | Uridine-2’-monophosphate | ++ | +++ | ++ | +++ | ++ | F8 | D-cysteine | ++ | +++ | ++ | +++ | H8 | p-amino benzene sulfonic acid | ++ | +++ | ++ | +++ |
| B9 | Guanosine-3’-monophosphate | ++ | +++ | ++ | +++ | D9 | Uridine-3’-monophosphate | ++ | +++ | ++ | +++ | ++ | F9 | L-cysteinyl-glycine | ++ | +++ | ++ | +++ | H9 | Butane sulfonic acid | ++ | +++ | ++ | +++ |
| B10 | Guanosine-5’-monophosphate | ++ | +++ | ++ | +++ | D10 | Uridine-5’-monophosphate | ++ | +++ | ++ | +++ | ++ | F10 | L-cysteic acid | ++ | +++ | ++ | +++ | H10 | 2-hydroxyethane sulfonic acide | ++ | +++ | ++ | +++ |
| B11 | Guanosine-2’,3’-cyclic monophosphate | ++ | +++ | ++ | +++ | D11 | Uridine-2’,3’-cyclic monophosphate | ++ | +++ | ++ | +++ | ++ | F11 | Cysteamine | ++ | +++ | ++ | +++ | H11 | Methane sulfonic acid | ++ | +++ | ++ | +++ |
| B12 | Guanosine-3’,5’-cyclic monophosphate | ++ | +++ | ++ | +++ | D12 | Uridine-3’,5’-cyclic monophosphate | ++ | +++ | ++ | +++ | ++ | F12 | L-cysteine sulfinic acid | ++ | +++ | ++ | +++ | H12 | Tetramethylene sulfone | ++ | +++ | ++ | +++ |

*: #Cond. stands for the assay conducted on the Biolog PM 4 plate. “−”, “+”, “++” and “+++” indicate that *B. cinerea* could not utilize the tested substrate or utilized the substrate poorly, moderately or effectively, respectively, on the Biolog PM 4 MicroPlate after 7 days of incubation at 28 °C. “V1”, “T1”, “C1” and “S1” denote the isolates of *B. cinerea* hosted by tomato, tobacco, cucumber and strawberry, respectively.

**S6 Table. Metabolic profiling of *Botrytis cinerea* hosted by tomato, tobacco, cucumber and strawberry on the PM 9 MicroPlate ***

| **#Cond.** | **Substrate** | **V1** | **T1** | **C1** | **S1** | **#Cond.** | **Substrate** | **V1** | **T1** | **C1** | **S1** | **#Cond.** | **Substrate** | **V1** | **T1** | **C1** | **S1** | **#Cond.** | **Substrate** | **V1** | **T1** | **C1** | **S1** |
| --- | --- | --- | --- | --- | --- | --- | --- | --- | --- | --- | --- | --- | --- | --- | --- | --- | --- | --- | --- | --- | --- | --- | --- |
| A1 | 1% NaCl | +++ | +++ | +++ | +++ | C1 | 6% NaCl + KCl | +++ | +++ | +++ | +++ | E1 | 1% Sodium Formate | +++ | +++ | +++ | +++ | G1 | 20 mM Sodium Phosphate pH 7 | +++ | +++ | +++ | +++ |
| A2 | 2% NaCl | +++ | +++ | +++ | +++ | C2 | 6% NaCl + L-Proline | +++ | +++ | +++ | +++ | E2 | 2% Sodium Formate | +++ | +++ | +++ | +++ | G2 | 50 mM Sodium Phosphate pH 7 | +++ | +++ | +++ | +++ |
| A3 | 3% NaCl | +++ | +++ | +++ | +++ | C3 | 6% NaCl + N-Acetyl-L-glutamine | +++ | +++ | +++ | +++ | E3 | 3% Sodium Formate | +++ | +++ | +++ | +++ | G3 | 100 mM Sodium Phosphate pH 7 | +++ | +++ | +++ | +++ |
| A4 | 4% NaCl | +++ | +++ | +++ | +++ | C4 | 6% NaCl + β-Glutamic acid | +++ | +++ | +++ | +++ | E4 | 4% Sodium Formate | +++ | +++ | +++ | +++ | G4 | 200 mM Sodium Phosphate pH 7 | +++ | +++ | +++ | +++ |
| A5 | 5% NaCl | +++ | +++ | +++ | +++ | C5 | 6% NaCl + γ–Amino-N-butyric acid | +++ | +++ | +++ | +++ | E5 | 5% Sodium Formate | +++ | +++ | + | +++ | G5 | 20 mM Sodium Benzoate pH 5.2 | +++ | +++ | - | +++ |
| A6 | 5.5% NaCl | +++ | +++ | +++ | +++ | C6 | 6% NaCl + Glutathione | +++ | +++ | +++ | +++ | E6 | 6% Sodium Formate | +++ | +++ | + | +++ | G6 | 50 mM Sodium Benzoate pH 5.2 | - | - | - | - |
| A7 | 6% NaCl | +++ | +++ | +++ | +++ | C7 | 6% NaCl + Glycerol | +++ | +++ | +++ | +++ | E7 | 2% Urea | +++ | +++ | + | +++ | G7 | 100 mM Sodium Benzoate pH 5.2 | - | - | - | - |
| A8 | 6.5% NaCl | +++ | +++ | +++ | +++ | C8 | 6% NaCl + Trehalose | +++ | +++ | +++ | +++ | E8 | 3% Urea | + | + | - | + | G8 | 200 mM Sodium Benzoate pH 5.2 | - | - | - | - |
| A9 | 7% NaCl | +++ | +++ | +++ | +++ | C9 | 6% NaCl + Trimethylamine-N-oxide | +++ | +++ | +++ | +++ | E9 | 4% Urea | - | - | - | - | G9 | 10 mM Ammonium Sulfate pH 8 | +++ | +++ | +++ | +++ |
| A10 | 8% NaCl | +++ | +++ | +++ | +++ | C10 | 6% NaCl + Trimethylamine | +++ | +++ | +++ | +++ | E10 | 5% Urea | - | - | - | - | G10 | 20 mM Ammonium Sulfate pH 8 | +++ | +++ | +++ | +++ |
| A11 | 9% NaCl | +++ | +++ | +++ | +++ | C11 | 6% NaCl + Octopine | +++ | +++ | +++ | +++ | E11 | 6% Urea | - | - | - | - | G11 | 50 mM Ammonium Sulfate pH 8 | +++ | +++ | +++ | +++ |
| A12 | 10% NaCl | +++ | +++ | +++ | +++ | C12 | 6% NaCl + Trigonelline | +++ | +++ | +++ | +++ | E12 | 7% Urea | - | - | - | - | G12 | 100 mM Ammonium Sulfate pH 8 | +++ | +++ | +++ | +++ |
| B1 | 6% NaCl | +++ | +++ | +++ | +++ | D1 | 3% Potassium Chloride | +++ | +++ | +++ | +++ | F1 | 1% Sodium Lactate | +++ | +++ | +++ | +++ | H1 | 10 mM Sodium Nitrate | +++ | +++ | +++ | +++ |
| B2 | 6% NaCl + Betaine | +++ | +++ | +++ | +++ | D2 | 4% Potassium Chloride | +++ | +++ | +++ | +++ | F2 | 2% Sodium Lactate | +++ | +++ | +++ | +++ | H2 | 20 mM Sodium Nitrate | +++ | +++ | +++ | +++ |
| B3 | 6% NaCl + N-N Dimethyl glycine | +++ | +++ | +++ | +++ | D3 | 5% Potassium Chloride | +++ | +++ | +++ | +++ | F3 | 3% Sodium Lactate | +++ | +++ | +++ | +++ | H3 | 40 mM Sodium Nitrate | +++ | +++ | +++ | +++ |
| B4 | 6% NaCl + Sarcosine | +++ | +++ | +++ | +++ | D4 | 6% Potassium Chloride | +++ | +++ | +++ | +++ | F4 | 4% Sodium Lactate | +++ | +++ | +++ | +++ | H4 | 60 mM Sodium Nitrate | +++ | +++ | +++ | +++ |
| B5 | 6% NaCl + Dimethyl sulphonyl propionate | +++ | +++ | +++ | +++ | D5 | 2% Sodium Sulfate | +++ | +++ | +++ | +++ | F5 | 5% Sodium Lactate | +++ | +++ | +++ | +++ | H5 | 80 mM Sodium Nitrate | +++ | +++ | +++ | +++ |
| B6 | 6% NaCl + MOPS | +++ | +++ | +++ | +++ | D6 | 3% Sodium Sulfate | +++ | +++ | +++ | +++ | F6 | 6% Sodium Lactate | +++ | +++ | +++ | +++ | H6 | 100 mM Sodium Nitrate | +++ | +++ | +++ | +++ |
| B7 | 6% NaCl + Ectoine | +++ | +++ | +++ | +++ | D7 | 4% Sodium Sulfate | +++ | +++ | +++ | +++ | F7 | 7% Sodium Lactate | +++ | +++ | +++ | +++ | H7 | 10 mM Sodium Nitrite | +++ | +++ | +++ | +++ |
| B8 | 6% NaCl + Choline | +++ | +++ | +++ | +++ | D8 | 5% Sodium Sulfate | +++ | +++ | +++ | +++ | F8 | 8% Sodium Lactate | +++ | +++ | +++ | +++ | H8 | 20mM Sodium Nitrite | +++ | + | +++ | +++ |
| B9 | 6% NaCl + Phosphorylcholine | +++ | +++ | +++ | +++ | D9 | 5% Ethylene Glycol | +++ | +++ | +++ | +++ | F9 | 9% Sodium Lactate | +++ | +++ | +++ | +++ | H9 | 40mM Sodium Nitrite | ++ | + | ++ | ++ |
| B10 | 6% NaCl + Creatine | +++ | +++ | +++ | +++ | D10 | 10% Ethylene Glycol | +++ | +++ | +++ | +++ | F10 | 10% Sodium Lactate | +++ | +++ | +++ | +++ | H10 | 60mM Sodium Nitrite | ++ | + | + | + |
| B11 | 6% NaCl + Creatinine | +++ | +++ | +++ | +++ | D11 | 15% Ethylene Glycol | +++ | +++ | +++ | +++ | F11 | 11% Sodium Lactate | +++ | +++ | +++ | +++ | H11 | 80mM Sodium Nitrite | ++ | + | + | + |
| B12 | 6% NaCl + L-Carnitine | +++ | +++ | +++ | +++ | D12 | 20% Ethylene Glycol | +++ | +++ | +++ | +++ | F12 | 12% Sodium Lactate | +++ | +++ | +++ | +++ | H12 | 100mM Sodium Nitrite | **++** | + | + | + |

*:#Cond. stands for the assay conducted on the Biolog PM 9 plate. “−”, “+”, “++” and “+++” indicate that *B. cinerea* could not utilize the tested substrate or utilized the substrate poorly, moderately or effectively, respectively, on the Biolog PM 9 MicroPlate after 7 days of incubation at 28 °C. “V1”, “T1”, “C1” and “S1” denote the isolates of *B. cinerea* hosted by tomato, tobacco, cucumber and strawberry, respectively.

**S7 Table. Metabolic profiling of *Botrytis cinerea* hosted by tomato, tobacco, cucumber and strawberry on the PM 10 MicroPlate ***

| **#Cond.** | **Substrate** | **V1** | **T1** | **C1** | **S1** | **#Cond.** | **Substrate** | **V1** | **T1** | **C1** | **S1** | **#Cond.** | **Substrate** | **V1** | **T1** | **C1** | **S1** | **#Cond.** | **Substrate** | **V1** | **T1** | **C1** | **S1** |
| --- | --- | --- | --- | --- | --- | --- | --- | --- | --- | --- | --- | --- | --- | --- | --- | --- | --- | --- | --- | --- | --- | --- | --- |
| A1 | pH 3.5 | +++ | +++ | +++ | +++ | C1 | pH 4.5 + L-Methionine | +++ | +++ | +++ | +++ | E1 | pH 9.5 | - | - | - | - | G1 | pH 9.5+Anthranilic acid | - | - | - | - |
| A2 | pH 4 | +++ | +++ | +++ | +++ | C2 | pH 4.5 + L-Phenylalanine | +++ | +++ | +++ | +++ | E2 | pH9.5+L-Alanine | - | - | - | - | G2 | pH 9.5 +L-Norleucine | - | - | - | - |
| A3 | pH 4.5 | +++ | +++ | +++ | +++ | C3 | pH 4.5 + L-Proline | +++ | +++ | +++ | +++ | E3 | pH9.5+L-Arginine | - | - | - | - | G3 | pH 9.5+L-Norvaline | - | - | - | - |
| A4 | pH 5 | +++ | +++ | +++ | +++ | C4 | pH 4.5 + L-Serine | +++ | +++ | +++ | +++ | E4 | pH9.5+L-Asparagine | - | - | - | - | G4 | pH 9.5+Agma tine | - | - | - | - |
| A5 | pH 5.5 | +++ | +++ | +++ | +++ | C5 | pH 4.5 + L-Threonine | +++ | +++ | +++ | +++ | E5 | pH9.5+L-Aspartic Acid | - | - | - | - | G5 | pH 9.5 +Cadaverine | - | - | - | - |
| A6 | pH 6 | +++ | +++ | +++ | +++ | C6 | pH 4.5 + L-Tryptophan | +++ | +++ | +++ | +++ | E6 | pH9.5+L-Glutamic Acid | - | - | - | - | G6 | pH 9.5+Putrescine | - | - | - | - |
| A7 | pH 7 | +++ | +++ | +++ | +++ | C7 | pH 4.5 + L-Tyrosine | +++ | +++ | +++ | +++ | E7 | pH9.5+L-Glutamine | - | - | - | - | G7 | pH 9.5+Histamine | - | - | - | - |
| A8 | pH 8 | +++ | +++ | +++ | +++ | C8 | pH 4.5 + L-Valine | +++ | +++ | +++ | +++ | E8 | pH9.5+Glycine | - | - | - | - | G8 | pH 9.5+Phe nylethy lamine | - | - | - | - |
| A9 | pH 8.5 | +++ | +++ | + | + | C9 | pH 4.5 + Hydroxy-L-Proline | +++ | +++ | +++ | +++ | E9 | pH9.5+L-Histidine | - | - | - | - | G9 | pH 9.5+Tyra mine | - | - | - | - |
| A10 | pH 9 | - | - | - | + | C10 | pH 4.5 + L-Ornithine | +++ | +++ | +++ | +++ | E10 | pH9.5+L-Isoleucine | - | - | - | - | G10 | pH 9.5+Crea tine | - | - | - | - |
| A11 | pH 9.5 | - | - | - | - | C11 | pH 4.5 + L-Homoarginine | +++ | +++ | +++ | +++ | E11 | pH9.5+L-Leucine | - | - | - | - | G11 | pH 9.5+Trimethylamine-N-oxide | - | - | - | - |
| A12 | pH 10 | - | - | - | - | C12 | pH 4.5 + L-Homoserine | +++ | +++ | +++ | +++ | E12 | pH9.5+L-Lysine | - | - | - | - | G12 | pH 9.5+Urea | - | - | - | - |
| B1 | pH 4.5 | +++ | +++ | +++ | +++ | D1 | pH 4.5 + Anthranilic acid | - | - | - | - | F1 | pH9.5+L-Methionine | - | - | - | - | H1 | X-Caprylate | ++ | + | - | - |
| B2 | pH 4.5 + L-Alanine | +++ | +++ | +++ | +++ | D2 | pH 4.5 + L-Norleucine | +++ | +++ | +++ | +++ | F2 | pH9.5+L-Phenylalanine | - | - | - | - | H2 | X-α-D-Glucoside | - | - | - | - |
| B3 | pH 4.5 + L-Arginine | +++ | +++ | +++ | +++ | D3 | pH 4.5 + L-Norvaline | +++ | +++ | +++ | +++ | F3 | pH9.5+L-Proline | - | - | - | - | H3 | X-β-D-Glucoside | - | - | - | - |
| B4 | pH 4.5 + L-Asparagine | +++ | +++ | +++ | +++ | D4 | pH 4.5 +α- Amino-N-butyric acid | +++ | +++ | +++ | +++ | F4 | pH9.5+LL-Serine | - | - | - | - | H4 | X-α-D-Galactoside | - | - | - | - |
| B5 | pH 4.5 + L-Aspartic Acid | +++ | +++ | +++ | +++ | D5 | pH 4.5 +p-Aminobenzoate | +++ | +++ | +++ | +++ | F5 | pH9.5+L-Threonine | - | - | - | - | H5 | X-β-D-Galactoside | - | - | - | - |
| B6 | pH 4.5 + L-Glutamic Acid | +++ | +++ | +++ | +++ | D6 | pH 4.5 + L-cysteic acid | +++ | +++ | +++ | +++ | F6 | pH9.5+L-Tryptophan | - | - | - | - | H6 | X-α-D-Glucuronide | - | - | - | - |
| B7 | pH 4.5 + L-Glutamine | +++ | +++ | +++ | +++ | D7 | pH 4.5 + D-Lysine | +++ | +++ | +++ | +++ | F7 | pH9.5+L-Tyrosine | - | - | - | - | H7 | X-β-D-Glucuronide | - | - | - | - |
| B8 | pH 4.5 + Glycine | +++ | +++ | +++ | +++ | D8 | pH 4.5 + 5-HydroxyLysine | +++ | +++ | +++ | +++ | F8 | pH9.5+L-Valine | - | - | - | - | H8 | X-β-D-Glucosaminide | - | - | - | - |
| B9 | pH 4.5 + L-Histidine | +++ | +++ | +++ | +++ | D9 | pH 4.5 + 5-HydroxyTryptophan | +++ | +++ | +++ | +++ | F9 | pH9.5+L-Hydroxy-L-Proline | - | - | - | - | H9 | X-β-D-Galactosaminide | - | - | - | - |
| B10 | pH 4.5 + L-Isoleucine | +++ | +++ | +++ | +++ | D10 | pH 4.5 + D,L Diamino-Pimelic acid | +++ | +++ | +++ | +++ | F10 | pH9.5+L-Ornithine | - | - | - | - | H10 | X-α-D-Mannoside | - | +++ | - | - |
| B11 | pH 4.5 + L-Leucine | +++ | +++ | +++ | +++ | D11 | pH 4.5 + Trimethylamine-N-oxide | +++ | +++ | +++ | +++ | F11 | pH9.5+L-Homoarginine | - | - | - | - | H11 | X-PO4 | + | +++ | - | - |
| B12 | pH 4.5 + L-Lysine | +++ | +++ | +++ | +++ | D12 | pH 4.5 + Urea | +++ | +++ | +++ | +++ | F12 | pH9.5+L-Homoserine | - | - | - | - | H12 | X-SO4 | +++ | +++ | - | - |

*: #Cond. stands for the assay conducted on the Biolog PM 10 plate. “−”, “+”, “++” and “+++” indicate that *B. cinerea* could not utilize the tested substrate or utilized the substrate poorly, moderately or effectively, respectively, on the Biolog PM 10 MicroPlate after 7 days of incubation at 28 °C. “V1”, “T1”, “C1” and “S1” denote the isolates of *B. cinerea* hosted by tomato, tobacco, cucumber and strawberry, respectively.
